# Methodological approaches in 16S sequencing of female reproductive tract in fertility patients: a review

**DOI:** 10.1007/s10815-024-03292-6

**Published:** 2024-10-21

**Authors:** I. M. Davidson, E. Nikbakht, L. M. Haupt, K. J. Ashton, P. J. Dunn

**Affiliations:** 1 Health Sciences & Medicine, Bond University, Gold Coast, Australia; 2 Stem Cell and Neurogenesis Group, Genomics Research Centre, Centre for Genomics and Personalised Health, School of Biomedical Sciences, Queensland University of Technology (QUT), 60 Musk Ave., Kelvin Grove, Brisbane, QLD 4059 Australia; 3 Centre for Biomedical Technologies, Queensland University of Technology (QUT), 60 Musk Ave., Kelvin Grove, Brisbane, QLD 4059 Australia; 4 ARC Training Centre for Cell and Tissue Engineering Technologies, Queensland University of Technology (QUT), Brisbane, Australia; 5 Max Planck Queensland Centre for the Materials Sciences of Extracellular Matrices, Queensland University of Technology (QUT), Brisbane, Australia

**Keywords:** ART (assisted reproduction technologies), IVF (in vitro fertilisation), 16S rRNA, NGS (next-generation sequencing), Bioinformatics

## Abstract

**Background:**

The female genital tract microbiome has become a particular area of interest in improving assisted reproductive technology (ART) outcomes with the emergence of next-generation sequencing (NGS) technology. However, NGS assessment of microbiomes currently lacks uniformity and poses significant challenges for accurate and precise bacterial population representation.

**Objective:**

As multiple NGS platforms and assays have been developed in recent years for microbiome investigation—including the advent of long-read sequencing technologies—this work aimed to identify current trends and practices undertaken in female genital tract microbiome investigations.

**Results:**

Areas like sample collection and transport, DNA extraction, 16S amplification vs. metagenomics, NGS library preparation, and bioinformatic analysis demonstrated a detrimental lack of uniformity. The lack of uniformity present is a significant limitation characterised by gap discrepancies in generation and interpretation of results. Minimal consistency was observed in primer design, DNA extraction techniques, sample transport, and bioinformatic analyses.

**Conclusion:**

With third-generation sequencing technology highlighted as a promising tool in microbiota-based research via full-length 16S rRNA sequencing, there is a desperate need for future studies to investigate and optimise methodological approaches of the genital tract microbiome to ensure better uniformity of methods and results interpretation to improve clinical impact.

**Graphical Abstract:**

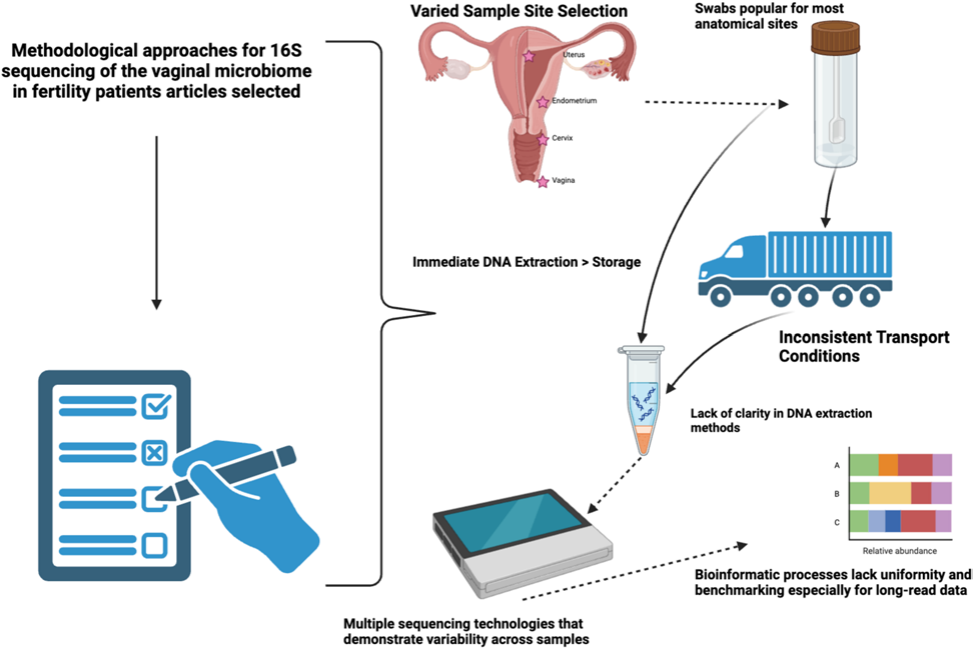

## Introduction

Infertility is defined as failure to fall pregnant following 12 months of unprotected sexual intercourse [1]. However, for women over 35 years of age, infertility can be diagnosed at six months [2]. Causes of infertility are many and varied and can involve male or female factors or a combination of both factors. Male factors involve sperm abnormalities, including decreased concentration, reduced motility, and irregular morphology [3]. For females, common causes of infertility can be linked to defects in ovulation, transport of ovum, implantation, and tubule diseases [4, 5]. Lifestyle factors such as smoking, obesity or being underweight, alcohol use, poor diet, and strenuous exercise also contribute to infertility in both males and females [6, 7]. Researchers have recently begun hypothesising whether the vaginal microbiome may also contribute to infertility.

The vaginal microbiome is pivotal in maintaining and promoting homeostasis in the vagina whilst out-competing pathogenic bacteria [8]. It is a dynamic environment that changes with age, pregnancy, hormones, menstruation, sexual activity, hygiene practices, and diseases that alter the microbial composition [9]. Due to the dynamic nature of microbiota, there is no definition of a ‘normal’ microbiome and the term healthy is used. This is typically defined by good pregnancy outcomes and the absence of disease and various infections [10]. A healthy vaginal microbiome interacts mutualistically with the host and bacteria by exchanging nutrients and shelter for competition and protection [11]. The host provides a humid and warm environment for the bacteria, whilst the native microbiota manufactures antimicrobial and anti-inflammatory factors for an initial line of defence against pathogenic bacteria [12].

Just as having a ‘healthy’ vaginal microbiome is pivotal for reproductive health, dysbiosis can have an impact on the results of assisted reproduction technology (ART) treatments, negatively impacting ART outcomes [13]. An example of this has been seen where inflammatory infections like bacterial vaginosis have been correlated with preclinical pregnancy loss. The presence of abnormal flora or bacterial vaginosis-associated microbes are commonly associated with infertility with a systematic review performed by van Oostrum et al. found 20% of infertile patients present with bacterial vaginosis-associated microbes and 39% of infertile patients present with abnormal flora [14]. Furthermore, women with bacterial vaginosis have a higher risk of pregnancy loss during in vitro fertilisation-embryo transfer (IVF-ET) cycles along with a decreased implantation rate, particularly if pathogenic microbes like *Chlamydia* are present [15]. Despite this, women with previous bacterial vaginosis prior to IVF have comparable pregnancy rates to women with no history of infections [16]. Interestingly, Kong et al. found that the increased incidence of *Gardnerella vaginalis* and *Atopobium vaginae* caused a failure to fall pregnant following embryo transfer or patients experienced a miscarriage [17]. Infections ascending from the vagina into the upper genital tract, particularly by *G. vaginalis* and *A. vaginae*, have been suggested as a major contributor to why biochemical pregnancy is less likely in women with an abnormal microbiome.

With a link between the female genital tract microbiome and reproductive outcomes, testing is needed to determine microbiome composition prior to subsequent procedures. Testing such as Nugent scoring and traditional culture methods require considerable effort, are time-consuming, and face bias in the selectivity of growth rates [18]. For this reason, molecular-based techniques such as next-generation sequencing (NGS) have rapidly become the gold standard for microbiome analysis [10, 18, 19]. More recently, the application of NGS has enhanced our understanding of the human microbiome composition [20]. NGS offers a remarkable depth and high-throughput accumulation of sequence information rapidly assessed from a single sample. NGS also allows for the classification and identification of microbes not identifiable through traditional culture methods, allowing the identification of new unique species.

Just as sequencing methods can influence the diversity of results, DNA extraction methods can also heavily influence the microbial diversity identified from a sample. DNA extraction methods remain the most time-consuming and cause of experimental variability in 16S rRNA NGS analysis, despite recent improvements to reduce errors and biases from the datasets [21]. Many factors influence variability between DNA extraction methods, including cell wall structure, reagent contamination, type of extraction (manual or automatic), and differences between laboratory staff and instrumentation used [22].

This review aims to critically evaluate the methodological approaches used in testing the female reproductive tract in patients undergoing fertility treatment, from the initial sampling site and collection technique through to the bioinformatic tools and pipeline development for analysis and interpretation of sequencing data. We carefully examine the associated bias, limitations, and advantages of each stage of the methodological approach to provide an optimal and uniform procedural approach for sequencing the genital tract microbiome in patients undergoing fertility treatment. In providing this approach, this review also aims to influence subsequent investigations and testing of the female reproductive tract in patients undergoing fertility treatment to inform and improve subsequent clinical practices and outcomes.

## Search strategies

An online search was performed using PubMed and google scholar databases of all international publications containing ‘microbiome OR microbiota OR microflora’ AND ‘infertility OR fertility OR sterility OR assisted reproduction OR recurrent implantation failure OR IVF OR in vitro fertilisation’ AND ‘next generation sequencing OR NGS OR 16S’ AND ‘reproductive tract OR genital tract OR vagina OR uterus OR endometrial OR cervical OR urogenital’. Studies were then assessed and used based on inclusion and exclusion criteria (Table 1).

**Table 1:** Inclusion and exclusion criteria used for selecting articles to be used in the review of 16S rRNA sequencing of the female reproductive tract in patients undergoing fertility treatment

	Inclusion criteria	Exclusion criteria
Publishing date	Studies published from 2018 onwards (excluding articles in Vitale et al. study)	Studies published before 2018 (excluding NGS papers in Vitale et al. study)
Specimen type	Human	Non-human
Patient characteristics	Female and undergoing any form of fertility treatment	Non-females and females not undergoing fertility treatment
Anatomical region	Studies assessing the human vaginal, cervical, uterine, or endometrial microbiome	Studies assessing microbiomes that are not vaginal, endometrial, cervical, or uterine
Sequencing methodology	Short-read or long-read NGS sequencing	Testing techniques that are not NGS
Sequencing approach	16S or metagenomics	Non-NGS sequencing

Only experimental types of research were selected in the PubMed search with reviews (including narrative or systematic reviews) excluded. Inclusion criteria included studies published from 2018 onwards (excluding articles in Vitale et al. paper); studies assessing the human vaginal, cervical, or endometrial microbiome via short-read or long-read sequencing; and studies using a 16S or metagenomics approach. Exclusion criteria included non-human studies, studies published before 2018 (excluding NGS papers in Vitale et al. study); studies assessing microbiomes that are not vaginal, endometrial, cervical, or uterine; and testing techniques that are not NGS. Studies published prior to the 2018 Vitale et al. study were excluded as they had provided some analysis on methodological approaches for DNA sequencing. However, as there was no conclusion provided on optimal methodological approaches, it was thought that an updated review was required. By completing the search strategy from 2018 to 2024, this yielded 29 studies that fit the aim of this review where we compared the methodologies employed for sampling, transport and storage, DNA extractions, and sequencing protocols from each original research article (Table 2).

**Table 2:** Summary of all included articles identified highlighting the methodologies and key microorganisms identified within each study

Authors	Sample site	Sampling method	DNA storage/transport method	DNA extraction	Sequencing approach	Sequencing technology	Bioinformatics method	Key microorganisms identified
Verstraelen et al. [26]	Endometrium	Endometrial biopsy and endometrial sampler brush	Samples stored at − 80 °C prior to transport and extraction	Pre-extraction lysis performed. DNA extraction method based off Vilchez-Vargas et al	V1-V2 region of 16S rRNA gene using 27F & 338R primers	Illumina technology	Mothur and R studio	*Bacteroidetes Pelomonas* genus, *Bacteroidetes xylanisolvebs*, *Bacteroidetes thetaiotaomicron*, *Bacteroidetes fragilis*, *Lactobacillus iners*, *Lactobacillus crispatus*, and *Prevotella amnii*
Franasiak et al. [68]	Endometrium	Tip of a transfer catheter	Cell-lysis performed and stored at − 20 °C prior to DNA extraction	DNA purification after lysis via bead beating	Metagenomics of 16S rRNA gene using Ion 16S™ Metagenomics Kit primer sets	Ion PGM ™ System	QIIME	*Flavobacterium* and *Lactobacillus* genus
Tao et al. [70]	Endometrium	Distal section of a transfer catheter	Lysis and extraction performed after sampling	Pre-extraction lysis. Bacterial genomic DNA Isolation kit (Norgen Biotek Corp, ON, Canada)	V4 region of 16S rRNA gene with Illumina V4 Workflow Primers	Illumina	QIIME	*Lactobacillus*, *Corynebacterium*, *Bifidobacterium*, *Staphylococcus*, and *Streptococcus*
Wee et al. [93]	Vagina, cervix, and endometrium	Swabs	Immediately transported and frozen at − 80 °C except for endometrial samples which incubated overnight at 4 °C and then frozen at − 80 °C	Enzymatic and mechanical lysis performed prior to DNeasy Blood and Tissue Kit (QIAGEN) used	V1-V3 regions of 16S rRNA using 27F & 534R and 27F Mix	Illumina	QIIME	*Gardnerella*, *Ureaplasma*, and *Lactobacillus*
Greenbaum et al. [9]	Cervix	Swabs	Stored at − 80 °C	Mechanical cell lysis then Powersoi® DNA Isolation Kit (MoBio	V3-V4 region of 16S rRNA gene with custom primers	Illumina	Mothur and R studio	*Lactobacillus*, *Gardnerella*, *Preveotella*, *Sneathia*
Kyono et al. [51]	Endometrium and vagina	Vaginal discharge and endometrial fluid	Samples put into DNA stabilising solution after collection	DNA extracted using Agencourt genfind v2 Blood & Serum DNA Isolation Kit	16S rRNA metagenomics	Illumina	StatMate V software	*Gardnerella*, *Streptococcus*, *Atopobium*, *Bifidobacterium*, *Sneathia*, *Prevotella*, and* Staphylococcus*
Kitaya et al. [73]	Endometrium and vagina	Endometrial fluid and vaginal swabs	Samples added to DNA stabilising solution	Pre-lysis performed with Proteinase K. Agencourt Genfind v2 Blood & Serum DNA Isolation Kit	V4 region of 16S rRNA gene using 515F and 806rB primers	Illumina	QIIME	*Lactobacillus*
Cheong et al. [67]	Endometrium	Swabs	Following DNA extraction, sample stored at − 20 °C	Mechanical lysis. DNA precipitated via isopropanol and sodium acetate	V3-V4 region metagenomics of 16S rRNA gene using Illumina provided primers	Illumina	Mothur	*Lactobacillus*, *Streptococcus*, *Veillonellaceae*, and* Preveotella*
Liu et al. [69]	Endometrium	Biopsy and fluid	Samples stored at − 80 °C prior to extraction	Extraction performed according to published protocol by Yuan et al. (Yuan et al., 2012)	V4 region of 16S rRNA gene only V4 primers specified	Illumina	Mothur	*Lactobacillus*, *Atopobium*, *Bifidobacterium*, *Preveotella*, and *Staphylococcus*
Bernabeu et al. [13]	Vagina	Swab	Samples stored at − 80 °C prior to extraction and − 20 °C after extraction	PureLink microbiome DNA purification kit (ThermoFisher)	V3-V4 region of 16S rRNA gene with 357F and 806R primers	Illumina	QIIME2	*Lactobacillus*, *Gardnerella*, *Streptococcus*, *Clostridium*, and *Ureaplasma*
Diaz-Martínez et al. [35]	Endometrium and vagina	Swabs	Frozen at − 80 °C after sampling	The PureLink Microbiome DNA Purification Kit (ThermoFisher)	16S rRNA metagenomics using 357F & 806R primers	Illumina	QIIME2	*Lactobacillus*, *Streptococcus*, *Preveotella*, *Ralastonia*, *Dialister*, and *Gardnerella*
Hao et al. [37]	Cervix	Swabs	Frozen at -80 °C after sampling	PureLink Microbiome DNA extraction Kit (ThermoFisher)	V3-V4 regions of the 16S rRNA gene using 341F & 806R primers	Illumina	QIIME	*Lactobacillus*, *Gardnerella*, *Desulfovibrio*, and *Preveotella*
Okwelogy et al. [52]	Vagina	Swabs	DNA preservation buffer	DNA extraction was based on a protocol developed by uBiome Inc	V4 region of 16S rRNA gene with 515F & 806R primers	Illumina	QIIME/UCLUST	*Lactobacillus*, *Preveotella*, and *Gardnerella*
Karaer et al. [38]	Vagina	Swabs	Swabs stored at − 80 °C	The QiAamp DNA Microbiome Kit (QIAGEN)	V3-V4 region of 16S rRNA gene with Gene-specific sequences were selected from the study by Klindworth et al. (Klindworth et al., 2013)	Illumina	QIIME2	*Lactobacillus*, *Gardnerella*, and *Streptococcus*
Ichiyama et al. [49]	Endometrium and vagina	Vaginal swabs and pipette insertion for endometrium samples	Samples immediately soaked in OMNIgene® VAGINAL	Agencourt genfind v2 Blood & Serum DNA Isolation Kit or MagNA Pure 24 Pathogen 200 hp 1.0 protocol	V4 region of 16S rRNA using 515f & 806rB primers	Illumina	QIIME	*Lactobacillus*, *Streptococcus*, *Gardnerella*, and *Preveotella*
Oberle et al. [71]	Endometrium	Leftover endometrial tissue from routine scratching	Sample stored at − 20 °C	The DNeasy Blood and Tissue Kit (QIAGEN) and PureLink™ Microbiome DNA Purification Kit (ThermoFisher) compared	V1-V9 of 16S rRNA gene using primers 27F & 1492R	Nanopore	EPI2ME	*Lactobacillus*
Lüth et al. [39]	Vagina	Swabs	Swabs frozen at -80 °C after sampling. Isolated DNA stored at − 20 °C	Mechanical lysis then DNeasy PowerSoil Kit (QIAGEN)	Both V3/V4 and V1-V9 16S rRNA gene using V3F & V4R and 27F-YM & 1492R-Y primers	Illumina (V3/V4) and Nanopore (V1-V9)	Mothur	*Lactobacillus*, *Haemophilus*, *Gardnerella*, *Preveotella*, and* Bifidobacterium*
Moreno et al. [40]	Endometrium	Endometrial fluid and biopsy	Samples transferred into RNAlater solution and stored at − 80 °C	Pre-digestion step included then QIAamp DNA Blood Mini Kit (QIAGEN)	16S rRNA metagenomics using primers according to the Ion 16S™ Metagenomics Kit (ThermoFisher)	Ion PGM™	QIIME2	*Gardnerella*, *Klebsiella*, *Lactobacillus*, *Streptococcus*, *Haemophilus*, *Neisseria*, *Chryseobacterium*, *Atopobium*, and *Bifidobacterium*
Keburiya et al. [83]	Uterus	Fragment of embryo transfer catheter	Sample placed in culture media and cultivated in an anaerobic box	DNA extraction was performed using PREP-RAPID DNA Extraction Kit	V3-V4 region of 16S rRNA gene using 357F & 806R primers	Illumina	QIIME	*Lactobacillus*, *Staphylococcus*, *Streptococcus*, *Gardnerella*, *Actinomyces*, *Bifidobacterium*, and* Candida*
Patel et al. [41]	Vagina	Swabs	Swabs stored at − 80 °C	DNA extraction was performed using QIAamp DNA Stool Mini Kit (QIAGEN)	V2-V3 region of 16S rRNA gene using 101F & 518R primers	Ion GeneStudio™ S5 system	QIIME2	*Lactobacillus*, *Leptotrichia*, *Snethia*, *Gardnerella*, and* Preveotella*
Reschini et al. [25]	Endometrium and vagina	Vaginal swab and endometrial fluid via aspiration with catheter	Both stored at − 80 °C	DNA was extracted via QIAamp DNA Microbiome kit (QIAGEN)	V3-V4-V6 regions of the 16S rRNA gene using Microbiota solution B kit and degenerative primers	Illumina	MicrobAT	*Lactobacillus*, *Gardnerella*, *Bifidobacterium*, *Propinibacterium*, *Pelomonas*, *Pseudomonas*, *Streptococcus*, and *Escherichia*
Villani et al. [53]	Cervix	Swabs	Swabs put into DNA stabilisation buffer. Following isolation, DNA was stored at − 80 °C	Mechanical lysis performed then DNA was extracted using QIAamp DNA Blood and Tissue kit (QIAGEN)	16S rRNA gene metagenomics with gene-specific sequences were selected from the study by Klindworth et al. (Klindworth et al., 2013)	Illumina	BBDUk	*Lactobacillus*, *Gardnerella*, *Atopobium*, *Bifidobacterium*, and *Streptococcus*
Vajpeyee et al. [42]	Vagina	Swabs	Swabs frozen at − 80 °C	DNA extracted via DNeasy PowerSoil kit (QIAGEN)	16S rRNA metageonimcs with 4-primer method PCR	Nanopore	MinKNOW/GSTK	*Lactobacillus*, *Gardnerella*, *Streptococcus*, *Atopobium*, *Preveotella*, *Snethia*, *Bifidobacterium*, and *Clostridium*
Komiya et al. [50]	Vagina	Vaginal lavage and swabs	Lavage samples stored at − 30 °C. Swabs stored at room temperature following a preservative liquid	DNA extracted via QIAamp UCP Pathogen Mini Kit (QIAGEN)	V1-V9, V3-V4 regions of the 16S rRNA gene via metagenomics using S-D-Bact-0008-c-S-20, 341F & 806R primers with anchor sequences on each	Nanopore	GUPPY/Minimap2	*Lactobacillus*, *Streptococcus*, *Atopobium*, and *Staphylococcus*
Zou et al. [43]	Endometrium	Endometrial tissue via endometrium sampler	Sample stored at − 80 °C	DNA extraction was performed using QIAamp DNA Microbiome Kit (QIAGEN)	V2, V4, V8 and V3, V6-7, V9 regions of the 16S rRNA gene via metagenomics using primers provided in 16S Ion Metagenomics Kit (ThermoFisher)	Ion S5 sequencing system and Ion 530 chip	QIIME2/Trimmomatic	*Lactobacillus*, *Gardnerella*, *Preveotella*, *Ralstonia*, *Anaerobacillus*, and *Streptococcus*
Ono et al. [32]	Uterine endometrium	Aspiration via an aspiration tube	Soaked in OMNIgene®- VAGINAL microbiome kit	Not stated	V4 using Illumina 16S metagenomic sequencing protocol	Illumina	JMP version 16	*Lactobacillus*, *Ureaplasma*, *Dialister*, and *Streptococcus*
Su et al. [85]	Vagina, cervix, and endometrium	Vaginal and cervical mucus swabs endometrial fluid through catheter	Samples stored at − 80 °C	TIANGEN Kit (G-BIO Bioinformatics Co., Ltd (DP316 China)	V3-V4 16S rRNA sequencing using 341F & 805R	Illumina	QIIME2	*Lactobacillus*, *Gardnerella*, *Sphingobium*, *Corynebacterium*, *Ralstonia*, *Enterobacter*, and *Enterococcus*
Nishio et al. [86]	Cervicovaginal mucus	Swab	Samples immediately stored at − 20 °C, then transferred to − 80 °C	ChargeSwitch Forensic DNA Purification Kit (Thermo Fisher Scientific, Waltham, MA, USA)	V3-V4 16S rRNA sequencing using Bakt_341F & Bakt_805R	Illumina	QIIME2	*Lactobacillus*, *Streptococcus*, *Gardnerella*, *Escherichia coli*, and *Bifidobacterium*
Wei et al. [87]	Endometrium	Catheter	Not stated	FastDNA Spin Kit (MP Biomedicals, Santa Ana, CA, USA)	V3-V4 16S rRNA sequencing using 338F & 806R	Illumina	QIIME2	*Rhodococcus*, *Pseudomonas*, *Archomobacter*, and *Lactobacillus*

## DNA preparation and influences on sequencing data

### Sampling methods

**Fig. 1:**
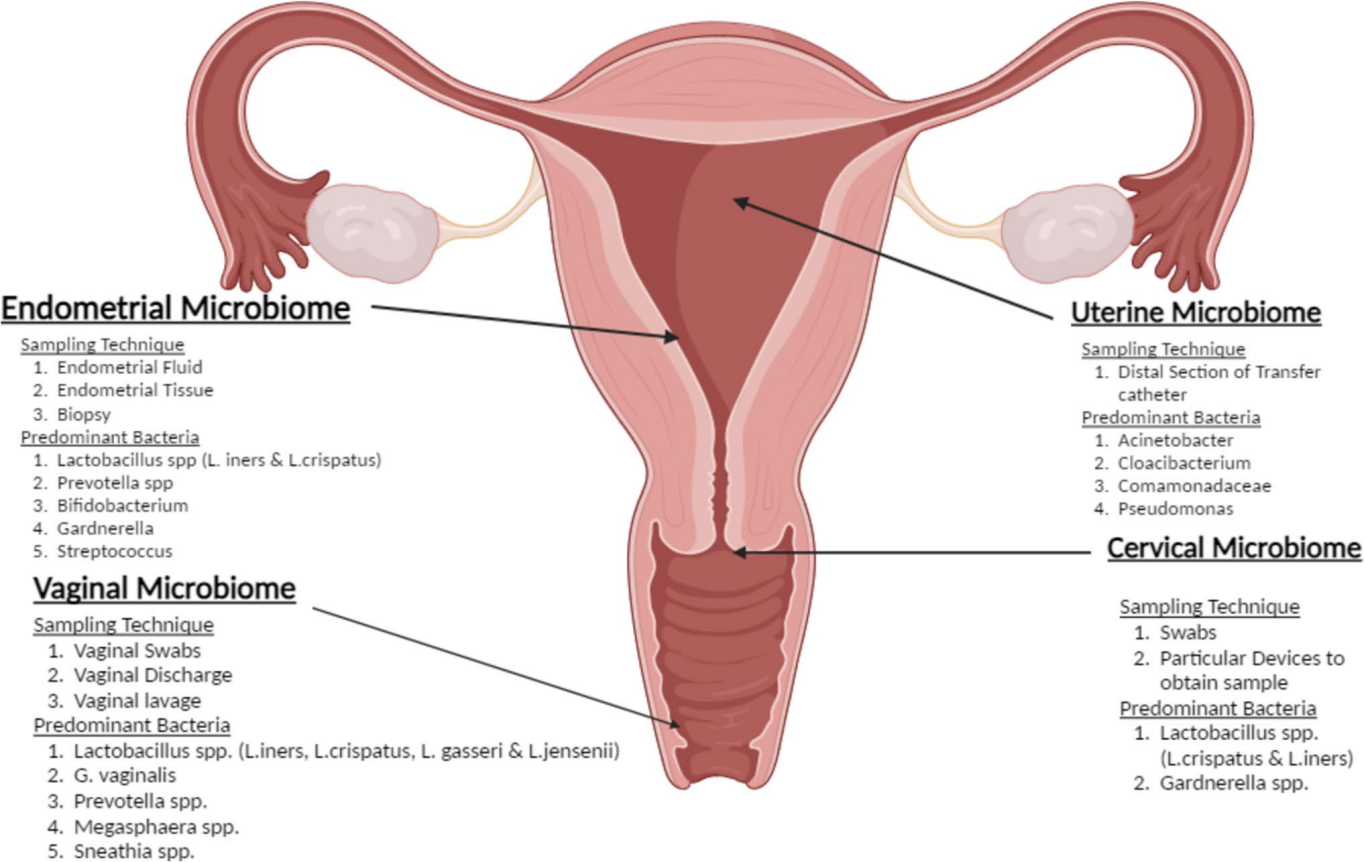
The most common sampling techniques and predominant bacteria found in each anatomical site of the female reproductive tract including the uterus, endometrium, cervix, and vagina for microbiome testing (image created in BioRender)

Unique microbial niches exist in different anatomical sites throughout the female reproductive tract with the sampling site influencing specific microbial species abundance and population representations [23]. As such, differing sampling methods—including sampling techniques and differing anatomical site selection—have demonstrated differing microbial population representation and microbial abundance results (Fig. 1) [24–26].

Previous studies investigating the vaginal microbiome used primarily vaginal swabs, with vaginal discharge and lavage samples used less commonly. Interestingly, although limited, data suggests differences in microbial composition in swabs, discharge, and lavage sampling for the vaginal microbiome [24]. In addition, comparison of swabbing types has also been compared, such as cytobrush and a swab, wherein the relative abundance of bacterial species was found to be very similar between both methods [24]. Additionally, several studies have recorded comparable bacterial abundances and diversity in self-collected versus clinically collected vaginal samples, supporting adequate sample collection without the requirement of a gynaecological examination [27, 28]. However, it is possible for microbes to be missed and variation to occur if the exact vaginal location is not disclosed following self-collection [29].

In cervical microbiome studies, swabs obtained from cervical and endocervical areas were the most represented, followed by specific devices used to obtain a transcervical sample. Interestingly, whilst there have been minimal comparisons to date examining cervical sampling techniques for microbiome composition, a cytobrush with an extended-tip spatula has been demonstrated as the best combination of collection for collecting endocervical cells for subsequent analysis [24]. This technique may also be suitable as the best practice for determining microbiome composition, but there was no mention of best practices concerning microbiome collection and minimisation of contamination from the vagina [24]. There must be significantly more studies investigating cervical microbiome collection techniques before a conclusion can be made on best practices.

Sample collections to investigate endometrial microbiome composition most commonly rely on endometrial fluid samples, followed by endometrial tissue from scratching and biopsies. Endometrial sampling poses significant challenges as contamination from the vagina and cervix is extremely difficult to avoid, with different sampling approaches returning significantly varied results regarding microbial content [24]. Endometrial biopsy samples considerably reduce the likelihood of contamination from the lower reproductive tract; however, they are highly invasive and usually obtained from patients with pre-existing conditions not representative of a healthy population [30]. Due to this, obtaining endometrial microbiome samples that lack contamination in a non-invasive way is extremely difficult. Reschini et al. suggest endometrial microbiome sampling via an embryo catheter in conjunction with rigorous aseptic methodologies to minimise contamination and invasiveness [25]. Determining any source of potential contamination is vital as this can significantly influence microbial community abundance, even for the same anatomical site.

Uterine microbiome samples collected via the distal section of a transfer catheter and the distal section of a transfer catheter are commonly used in embryo transfer or via aspiration. Until recently, the uterine microbiome was thought to be sterile, and little is still known about the microbiome of the uterus. Recent studies have identified microbes from uterine samples; however, caution is suggested for these findings with a high possibility of endometrial contamination likely influencing these results [26, 31, 32]. In patients not participating in ARTs, uterine samples are commonly obtained during hysterectomies and hysterectomy procedures [31]. However, for subsequent ART procedures, uterine microbiome samples are only obtainable through the distal section of a transfer catheter, posing significant potential contamination from vaginal, cervical, and endometrial microbes [26, 31]. Transcervical approaches are currently used, with further research and optimisation required to obtain pure uterine microbiome samples [26].

### Sample storage and transport

Delays in DNA extraction after sampling require samples to be stored effectively to ensure the composition of microbiota remains representative of the populations at the time of sampling. Frozen storage of samples (− 80 °C) is considered the ‘gold standard’ best practice to ensure preserved microbes and prevention of the microbiota from changing significantly [33, 34].

In terms of sample storage following collection across literature, the majority of microbiome composition samples were stored at either − 20 °C or − 80 °C. [13, 25, 35–43]. Whilst freezing bacterial samples has shown to alter the cellular structure of gram-positive bacteria, causing a higher ratio of Firmicutes to Bacteroidetes, the change was of no statistical significance [44]. To ensure the sample is the most representative population of the sample site, immediately freezing samples to − 80 °C is considered the ‘gold standard’ to ensure preservation of microbes and prevention of the microbiota changing significantly at room temperature (21–24 °C) [32, 45]. Prakash et al. (2020) identified that, generally, microbial samples stored at room temperature for an extended period resulted in changes in microbial communities and overall composition [46]. Choo et al. also found substantial microbe compositional changes in samples stored at room temperature when compared to samples stored at 4 °C or − 80 °C [43]. Whilst sample storage temperatures can significantly impact microbial composition, sample storage methods can also heavily influence alpha and beta diversity [47]. Alpha and beta diversity allow for the microbial differences within a sample (alpha) or across multiple samples (beta) to be quantified [48].

Additionally, some studies opt to add some form of DNA stabilising solution following sample collection prior to storage or bypassing freezing to maintain microbial composition of samples [40, 49–53]. The addition of DNA stabilising solutions before freezing have been found to preserve microbial DNA integrity, allowing for the transportation of microbiome samples [54]. However, different stabilising solutions can influence microbial alteration. As an example, Choo et al. found that samples stored in Tris–EDTA buffer showed the most significant bacterial change when compared to the non-commercial DNAgenotek produced OMNIgene.GUT that aims to optimise collection for nucleic acids [44, 55]. Samples stored in Tris–EDTA buffer demonstrated an increased operational taxonomic unit abundance of facultative anaerobes such as Proteobacteria and decreased abundance of *Firmicutes* and *Actinobacteria* [44]. The reduction in specifically *Firmicute* abundance would significantly impact female tract microbiome samples as the *Lactobacillus* genera results from the *Firmicutes/Bacillota* phylum and is not only the most abundant genera found but the biggest indicator of a healthy or dysbiotic environment [26, 56, 57]. It is also worth noting that the use of lysis buffers in sample storage has resulted in an overall reduction in DNA yield but not in DNA integrity [58]. Additionally, samples stored in an RNA preservation buffer such as RNA later have demonstrated significant changes in bacterial diversity when compared with storage alone at − 80 °C along with a reduced DNA extraction yield and purity [44].

Choosing to complete DNA extraction directly after sampling rather than DNA extraction directly following sample freezing has also shown different impacts on microbial composition. Vogtmann et al. found that compared to immediate DNA extraction, freezing microbial samples resulted in an increased abundance of the Firmicutes strain and a decreased abundance of Bacteriodetes microbes [59]. However, due to factors such as transport and time constraints, DNA extraction after sample storage is the most common method of choice [59].

Whilst sampling methods influence microbial composition, sampling methods are less influential over microbiome abundance and diversity than individual variation [60, 61]. Overall, it should be recommended that to enable the most accurate microbial representation and provide high DNA integrity and yield, it is best practise to immediately extract DNA from samples and store extracted DNA at − 80 °C. If DNA extraction is unable to be completed at that time, samples should be immediately frozen at samples at − 80 °C until DNA extraction can occur.

### DNA extraction methods

Just as sampling methods influence microbial composition, DNA extraction methods also impact the presence and abundance of certain microbes and vary significantly from study to study (Table 1). Choosing the correct DNA extraction method is vital as vaginal samples contain high proportions of host DNA (> 90%), which can impair species detection [62]. Specific DNA extraction techniques are believed to enhance the yield of gram-positive bacteria but may not be as effective for gram-negative bacteria, potentially misrepresenting the true population composition. Due to the increased rigidity and strength of gram-positive bacterial cell walls, more extensive lysis protocols are required to obtain their DNA. However, these rigorous methods can over-degrade gram-negative bacteria, thereby reducing their yields [63]. The inverse is also demonstrated when lysis techniques are unable to effectively lyse gram-positive bacteria, leading to decreased DNA yields and misrepresenting population diversity [63, 64]. However, efficient mycobiome lysis is dependent on repeated bead-beating and enzymatic lysis which are integral for fungal DNA extraction [65].

Interestingly, some results demonstrated the use of DNA extraction kits non-specific to microbiomes and including a pre-lysis step, on average, increased DNA yield and microbial diversity when compared to DNA extraction kits specific to microbiomes and lacking a pre-lysis step [66]. The use of optimised methodologies from previously published articles or custom protocols is the most common choice for DNA extraction [26, 35, 39, 42, 43, 71]. For studies using DNA extraction kits, the most commonly used kit used is the DNeasy PowerSoil kit, followed by other kits such as Agencourt Genfind v2 Blood & Serum isolation kit, PureLink Microbiota DNA extraction kit, and the QIAamp DNA Microbiome kit [26, 31, 35, 39, 42, 43, 71]. A comparative study by Mattei et al. recorded that the QIAGEN DNeasy method achieved a higher yield and quality than a modified MoBio PowerSoil kit. However, the MoBio PowerSoil kit demonstrated higher microbial diversity when compared to the DNeasy method and higher diversity than the standard protocol [72]. A summary of these comparisons is provided in Table 3 including the extraction technique, supplier, key steps used in template isolation, and the perceived advantages and limitations of the kits commonly used.

**Table 3:** Comparison of different DNA extraction techniques key steps, advantages, and limitations [39, 41, 43, 53]

Extraction technique	Key steps	Advantages	Limitations	References
DNeasy PowerSoil Kit (QIAGEN)	1. Samples lysed via chemical and mechanical homogenisation 2. Bead beating and inhibitor removal 3. Purified lysate mixed with DNA binding solution, filtered and washed 4. Silica-bound DNA eluted	Easy to replicate Increased purity of isolated DNA Removes contaminants efficiently Effective for highly contaminated samples like stool or soil	Aggressive lysis throughout protocol can cause fragmentation of DNA	[39, 53, 71, 88]
Agencourt Genfind v2 Blood & Serum Isolation Kit (Beckman Coulter)	1. Sample lysed via chemicals 2. Sample bound to paramagnetic beads 3. Beads separated from contaminants via washing DNA eluted	Easy to replicate protocol High DNA yield	It does not provide an expected concentration range for samples, and it can be unclear how much of a sample is required for DNA extraction	[49, 51, 72]
PureLink Microbiota Kit (Thermo Fisher Scientific)	1. DNA lysed via bead beating and heat 2. DNA bound to silica-based column 3. DNA washed in a silica-based column 4. DNA eluted	Easy to replicate and different protocols for different sample types Encompasses a wide variety of sample types It recovers DNA with high purity and eliminates inhibitory compounds Typical recovery is 0.1–5 µg DNA	Not high-throughput compatible	[13, 35, 37, 71]
QIAamp DNA Microbiome Kit (QIAGEN)	1. Host cells are gently lysed to leave bacterial cells intact 2. Released host DNA is enzymatically degraded via enzymes 3. Combination of mechanical and chemical lysis used to degrade bacteria cells 4. Bacterial DNA bound to the silica membrane 4. Membrane washed to remove contaminants 5. DNA eluted	Depletes host DNA to maximise bacterial DNA Allows for highly sensitive 16S rDNA-based microbiome analysis	Host DNA enzymatic degradation may degrade the DNA of bacteria released via initial lysis DNA purity decreased as contaminant removal is minimal	[25, 38, 43]

Additionally, some studies choose to complete a pre-digestion step before DNA extraction consisting of enzymatic and/or mechanical cell lysis to degrade bacterial cell walls for difficult-to-lyse bacteria in an effort to increase the overall yield of isolated DNA [51, 67, 68, 73, 74]. However, Gill et al. suggest that the choice of lysis method does not influence the detection of microbial communities yet do recommend a uniform method be applied to all samples [75]. Interestingly, DNA extraction methods that include bead beating have been demonstrated to improve microbial identification and representation [76, 77]. However, the use of bead beating has also been identified to increase contamination in samples [75].

Choosing automated versus manual DNA extraction practises can influence DNA purity, yield, and the representation of microbes in downstream analysis. Whilst robotic DNA extraction methods can prove to be time efficient and consistent across large sample sizes, their use is rarely tested and validated for non-human DNA extractions. The use of liquid handling robots throughout DNA extraction has shown to increase cross contamination from well-to-well plate-based DNA extractions than manual DNA extraction. To reduce this risk, it is suggested that additional positive and negative controls be established [78].

Unfortunately, the lack of consistency in DNA extraction methods for microbial studies across the literature makes it extremely difficult to compare results between studies and can lead to inaccurate conclusions. As a result, no specific DNA extraction kits can be recommended as superior in microbial analysis. However, it is recommended that DNA is extracted manually rather than a well-to-well robotic process to decrease cross contamination. The use of DNA extraction kits with a bead-beating step is also highly recommended to enable accurate microbiome representation and degradation of certain cell components along with the inclusion of positive and negative controls [22].

### Sequence methodologies and robustness

#### General overview of sequencing methods

16S rRNA sequencing is currently the most widespread method for determining microbiome composition in female reproductive tracts; however, some studies have highlighted the utility of using a metagenomics approach rather than amplicon sequencing. Amplicon sequencing entails amplifying a variable region of highly conserved genes such as the 16S ribosomal RNA (rRNA) gene in DNA via PCR, followed by NGS for species identification and quantification [79]. However, a metagenomic approach sequences all the DNA present within a sample for identification and quantification [80].

#### 16S sequencing

**Fig. 2:**
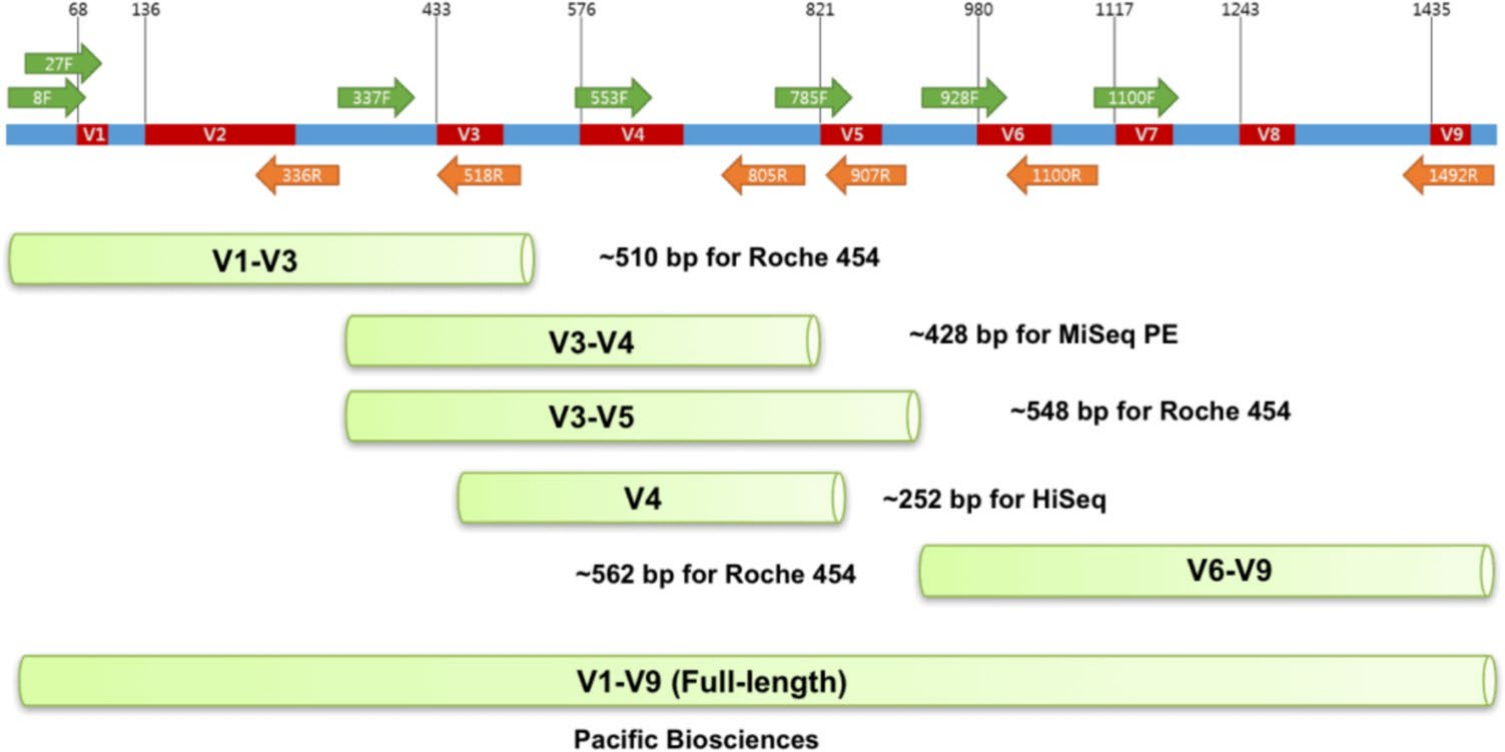
Structure of the 16S rRNA gene showing the conserved (blue) and nine hypervariable (red) regions. Popular primer sets (green, orange arrows) for different NGS platforms are shown—adapted with permission [82]

Amplicon sequencing performed by NGS offers remarkable depth and high-throughput information, with large quantities of sequence information rapidly assessed from a single sample. The selected amplicon for sequencing must be present in all microbes and contain regions of high conservation (to allow for consistent primer binding) in addition to regions of high variability to provide species-specific signatures. For this reason, the 16S rRNA gene is commonly used [79]. The 16S rRNA gene is approximately 1500 bp in size and has several highly conserved regions interleaved with nine hypervariable regions that demonstrate considerable sequence diversity amongst different microbial species and genera (Fig. 2) [81].

Many combinations exist for choice of 16S rRNA region to be used in investigations using amplicon sequencing. Targeting 1–2 hypervariable regions is the most popular length of choice with specifically the V3-V4 hypervariable regions amongst the most popular (Table 4) [13, 25, 35–39, 48, 51, 67, 83–87]. However, it is not uncommon for studies to examine a single hypervariable region or many pairs at once. For example, Kyono et al. investigated the V4, V1-V2, and V3-V4 hypervariable regions, whereas Tao et al. only investigated hypervariable region V2 [51, 70]. Regions V1, V2, and V3 are consistent throughout hypervariable region selection with the V1-V2 fragments of 16S rRNA possessing the highest resolution for species and genera taxa. Thus, choosing either of these regions for examination allows for a more specific separation of the sequencing data into species-level operational taxonomic units (OTUs) for subsequent downstream analysis [88, 89].

**Table 4:** Comparison of each hypervariable region chosen, the associated primer sequences, the limitations and advantages of hypervariable region selection and potential primer design, and reference

Region chosen	Primer	Primer sequences	Advantages	Limitations	Reference
V3-V4	**Forward**: 341F, 357F &515F **Reverse**: 806R	**Forward 341F**: 5′-CCTACGGGNGGCWGCAG-3′ **Forward 357F**: 5′-CCTACGGGAGGCAGCAG-3′ **Forward 515F:** 5′-GTGCCAGCMGCCGCGGTAA-3′ **Reverse**: 5′- GGACTACHVGGGTWTCTAAT-3′	Results in the highest richness and alpha diversity and provides the most extensive microbial population for partial 16S rRNA sequencing	Can result in low resolution for taxonomies causing a significantly lower discriminatory power	[13, 31, 35, 37–39, 51, 53, 67]
V4	**Forward**: 515F **Reverse**: 806R	**Forward**: 5′-GTGCCAGCMGCCGCGGTAA **Reverse**: 5′- GGACTACHVGGGTWTCTAAT-3′	Highly conserved region which can be easily targeted	Only one hypervariable region decreases resolution and decreases discriminatory power. Species level identification reduced due to high conservation	[51, 52, 69, 70, 73]
V1-V3	**Forward**: 27F **Reverse**: 515R	**Forward**: 5′-AGA GTT TGA TCC TGG CTC AG-3′ **Reverse**: A 5′-TTACCGCGGCTGCTGG -3′	Accurately identifies microbes and has a high taxonomic resolution for partial 16S rRNA sequencing	Can result in low resolution for taxonomies causing a significantly lower discriminatory power	[36, 93, 99]
V1-V2	**Forward**: 27F **Reverse**: 338R	**Forward**: 5′-AGA GTT TGA TCC TGG CTC AG-3′ **Reverse**: 5′-TGCTGCCTCCCGTAGGAGT-3′	Poses the highest level of resolution for species and genera for partial 16S rRNA sequencing	Can result in low resolution for taxonomies causing a significantly lower discriminatory power. Does not include the most conserved (V4) or most variable regions (V3)	[49, 51]
V1-V9	**Forward**: 27F & 27F-YM **Reverse**:1492R & 1492R-Y	**Forward 27F:** 5′-AGA GTT TGA TCC TGG CTC AG-3′ **Forward 27F-YM**: 5′AGAGTTTGATYMTGGCTCAG-3′ **Reverse 1492**: 5′-GGT TAC CTT GTT ACG ACT T-3′ **Reverse 1492R-Y**: 5′-GGTTACCTTGTTAYGACTT-3′	Allows for the whole 16S rRNA gene to be sequenced increasing resolution and discriminatory power	Primers required to sequence whole 16S rRNA gene (V1-V9), represent microbial populations inaccurately through misidentifying microbes	[39, 71]
V2-V3	**Forward**: 27F **Reverse**:338R	**Forward**: 5′-AGA GTT TGA TCC TGG CTC AG-3′ **Reverse**: 5′-TGCTGCCTCCCGTAGGAGT-3′	Poses a high level of resolution for species and genera for partial 16S rRNA sequencing	Doesn’t encompass all hypervariable regions decreasing taxonomic resolution and accurate microbial representation	[41]

Whilst not as popular as analysis of 1–2 hypervariable regions, Oberele et al. and Lüth et al. chose to analyse all hypervariable regions (V1–V9) [39, 71]. The choice of 1–2 hypervariable regions is most commonly used as the majority of NGS technologies are only capable of generating short read lengths, allowing for a small number of hypervariable regions to be investigated. However, this approach can create issues for accurate taxonomic classification and result in low resolution for taxonomies causing a significantly lower discriminatory power. In comparison to full-length 16S rRNA sequencing, the sequencing of 1–2 hypervariable regions also limits the ability to characterise microbial communities, across a potential nine hypervariable regions [20, 79, 90, 91].

Just as hypervariable region selection can be variable, as can primer design as shown in Table 3. When investigating hypervariable regions, V3-V4 primer pair 515F and 806R are commonly used with individual modifications made per study such as in Kitaya et al., where the primers were modified to give 515FB and 806RB where B distinguishes updated primers from older primers [73]. For those who examined all hypervariable regions—including Lüth et al. and Oberle et al.—primers 27F & 1492R were consistently used across these studies, with Lüth et al. slightly modifying the primers to act as degenerative primers, to allow for variability of bases during amplification [39, 71, 92].

Remarkably, Wee et al. modified the 27F primer by including a mix of seven other primers, including a four-oligo mix, *Bifidobacterium* specific sequence, *Borrelia* and *Chlamydia* sequence-specific sequences [93]. Unfortunately, the 27F primer demonstrated significant limitations in microbial detection and accurate representation, making it a significantly less robust choice than other primers, such as 515F. Lüth et al*.* highlight the limitations associated with the 27F primer which was particularly problematic and failed to identify *Chlamydia trachomatis* and overestimated *Lactobacillus iners*, significantly impacting the accuracy of microbial representations for clinical interpretation, making the 27F, a significantly less robust choice compared to other primers, such as 515F for microbial detection and representation [39].

Whilst similar primers are used and can be slightly altered, under-representation and over-representation of species within the sample pose a significant issue for studies assessing the pathophysiology and abundances of microbes in microbiome samples. The use of primers 27F and 1492R and subsequent modifications pose the most significant risk of this overestimation and underestimation of specific microbes due to nucleotide mismatches [39]. In a clinical setting, this can result in inaccurate treatment due to clinical misinterpretation, potentially causing harm to the patient or having no effect in treating the dysbiosis. Therefore, it is critical to design and use primers that equally report the microbes within samples to allow for the most accurate representation of patient microbiomes for subsequent analysis and clinical decisions.

#### Metagenomics

Metagenomic approaches can be used to identify different microbial populations without the inherent biases that arise from the amplification of a single gene by PCR and categorising the genes present in a microbial community via selective sequencing of all DNA extracted [94, 95]. The need for amplifying a single gene is removed in a metagenomic approach, allowing for vigorous microbial population estimations of composition and diversity [94]. The diversity and composition of microbial communities are analysed via data sequencing following the merging of the genomes of microflora [96]. However, as all DNA is indiscriminately sequenced, amplification can still demonstrate bias and a metagenomic approach demands an increase in the depth of sequencing, raising overall costs [80]. Whilst metagenomics allows for whole DNA sequencing, sequencing 1–2 hypervariable regions at a time via metagenomics remains a popular approach throughout female reproductive tract microbiome investigations using primers provided in metagenomics kits [35, 40, 43, 53, 67, 68, 74].

#### 16S vs. metagenomic sequencing approach

The utilisation of both amplicon sequencing and metagenomics have limitations, with microbial community identification with amplicon sequencing potentially introducing bias due to unequal amplification of the 16S rRNA genes of some species [84]. A 16S rRNA sequencing approach limits taxonomic resolution due smaller length of the amplicon product. Additionally, the 16S amplicon sequencing approach only allows for identification of bacteria and archaea, excluding fungi, viruses, and micro-eukaryotes. Despite these limitations, due to the small amplicon size of 16S rRNA sequencing, higher read depths are more easily obtained compared to metagenomic sequencing, which reduces the overall cost of this approach. Metagenomic sequencing also has some limitations, in that it relies more heavily on a reference database for assembly. This has been shown to reduce its capacity in identifying novel microbes within samples and increases the false positive likelihood whilst also demanding more computationally [80, 84, 95, 96]. Interestingly, both approaches have been demonstrated to give different community structures for microbial communities in samples obtained from multiple body sites [84] Whilst 16S rRNA sequencing proves a time and cost-efficient method, a metagenomics approach of sequencing microbial DNA within a sample is recommended to increase representation of bacteria, archaea, fungi, viruses, and micro-eukaryotes especially within the female reproductive microbiome [80, 96].

#### Comparison of the different NGS sequencing platforms

NGS technology encompassing short-read sequencing includes Illumina technology and Ion Torrent Systems, along with third-generation sequencing technologies (long-read sequencing) such as provided by nanopore and PacBio technology. All of these technologies allow for high-throughput sequencing whilst dramatically reducing costs per sample compared to more iterative sequencing methods such as Sanger sequencing.

#### Illumina-based studies

The NGS technology provided by Illumina sequences DNA via bridge amplification PCR, with DNA sequenced via the incorporation of fluorescent nucleotides to determine the DNA sequence. Illumina sequencing allows for sequencing to occur from both ends of a DNA fragment, termed paired-end sequencing, generating sequence data with in-depth coverage and high quality [97, 98].

In contrast to the high variability of hypervariable region selection, primer design and synthesis applied to the examination of microbiome samples, with Illumina sequencing, emerged as the most common method of choice [36, 38, 49, 51, 52, 70, 80, 99]. This can likely be attributed to the low error rate, reported accuracy of > Q30, and low DNA input (50–1000 ng) requirement of the Illumina NGS protocol [100, 101]. A quality score—or *Q*-score—represents the probability that a base is called wrong [102, 103]. For example, if the reported accuracy of Illumina sequencing is > Q30, that means that there is a 1 in 1000 or 0.001% chance the base was called incorrectly.

However, Illumina sequencing does demonstrate low taxonomic resolution and decreased accuracy in taxonomic classification due to the inability to sequence the whole 16S rRNA gene in one fragment. Although Illumina NGS is currently the most common method of choice, it is limited to a maximum of 600 bp (2 × 300 bp) read lengths per sequencing run. This only allows for a maximum of two of the nine hypervariable regions to be amplified in one fragment, impacting genus-level identification and creating discrepancies in diversity dependent upon the choice of regions [20, 79, 90, 91, 100].

#### Ion Torrent-based studies

The Ion Torrent NGS technology amplification relies on emulsion PCR, a bead-based method by which PCR occurs in micelles. The Ion Torrent technology utilises a semiconductor chip that contains a pH sensor to detect nucleotides via hydrogen ion release which enables read lengths between 200 and 600 bp and run times between 2 and 4 h. Ion Torrent systems are most suited to sequencing RNA and DNA templates of smaller size and the characterisation of small variants [98].

Compared to the multitude of studies using Illumina technology, research using Ion Torrent systems less abundant, with Illumina the most utilised NGS technology. However, studies using Ion Torrent technologies have recorded limited resolution of the 16S rRNA sequence and were unable to accurately identify specific bacterial species [41]. Whilst species-level identification was not obtained, the use of Ion Torrent systems has allowed for robust characterisation at genera-level identification [41, 68]. Moreno et al. found that the platform might detect DNA contamination skewing the microbiome community interpretation and composition [40]. Whilst Ion Torrent systems have a low error rate and sequence significantly faster than the Illumina platforms compared (up to 56 h), this technology is unable to recognise long repetitive sequences as well as large structural variants and assemble genomes [41, 97, 98, 100].

#### Third-generation sequencing-based studies

The emergence of third-generation sequencing platforms including Oxford Nanopore (nanopore sequencing) and Pacific Biosciences (PacBio sequencing) technologies have allowed for advancements in DNA sequencing, enabling sequences of over 10 kb in length to be generated and analysed [97, 98]. PacBio technology sequences DNA via a single-stranded circular DNA where a polymerase binds to an adapter sequence and amplifies DNA with fluorescent nucleotides, which, when bound, emit a light pulse interpreted as a nucleotide sequence [98]. Nanopore sequencing utilises a nanoscale protein pore (nanopore) which acts as a biosensor with DNA driven through the nanopore via a motor protein, disrupting an electric current, which is then decoded using algorithms to give real-time sequencing [104].

Unfortunately, no studies have yet investigated the microbiome of the female reproductive tract undergoing fertility treatment using PacBio sequencing. However, findings from a study assessing PacBio full-length sequencing for vaginal bacterial 16S rRNA gene classification by Wagner et al. recommended pooling no more than ten samples to enable data differentiation. Wagner et al. also found that high stringency post-screening on the PacBio platform removed true positive OTUs and therefore removed species from the dataset, with low stringency data containing additional false positive species [105].

Studies using nanopore technology have found a time and cost-efficient method for microbiota sequencing of the human vaginal microbiome with precise results. Additionally, nanopore sequencing demonstrated a rapid means for identifying microbes with a high resolution, enabling real-time analysis [39, 42, 50, 71]. In addition, nanopore sequencing has been highlighted as a potential feasible method for interventional studies to develop a portable microbiome-based clinical decision platform. This aligns with literature suggesting the potential for nanopore sequencing to contribute to clinical decision-making regarding ARTs [39]. However, the current 16S rRNA barcoding kit provided by ONT failed to identify bacteria including G. *vaginalis* and Bifidobacterium, decreasing overall resolution and an inaccurate determination of the microbial population representation [15]. Lüth et al. highlight the application of nanopore sequencing to species-level identification along with a reduction in false positives by adding an Emu algorithm [39]. The Emu algorithm creates microbial abundance profiles from full-length 16S rRNA sequencing reads by utilising an expectation–maximisation algorithm [106].

Overall, third-generation sequencing—specifically nanopore sequencing—has shown promise as a possible future microbiota-based diagnostic platform and potential therapeutic indicator for future research. The utilisation of third-generation sequencing in the clinical may enable clinicians to perform microbial analysis accurately as well as more efficiently and quickly. However, further in-depth investigations comparing methodological approaches for 16S sequencing of the female upper reproductive tract need to be performed to ensure a clinically robust methodology.

#### Bioinformatic pipeline tools and workflows

Bioinformatic pipeline usage and development vary significantly across the literature, with a multitude of tools available for each step and an abundance of data analysis options. Additionally, bioinformatic tools differ depending on which technology was selected for the study, with software compatible with only short-read lengths, or specifically tailored to long-read lengths.

#### Databases

To achieve accurate taxonomic classification, to provide precise population representation, reference sequence databases and their associated taxonomical classifications need to be carefully selected. The choice of reference database can significantly influence the accuracy of taxonomic classification results, particularly from understudied environments and outdated databases [107]. The Greengenes, NCBI, and SILVA databases are currently the most commonly used throughout microbial investigations [13, 31, 38, 40, 51, 71]. The Greengenes database provides full-length 16S rRNA curated taxonomy based on de novo tree inference and is regularly updated [108]. As such, the application of nanopore technologies to studies would demonstrate different microbial communities due to the limited compatible bioinformatic tools currently available. In contrast, the NCBI database uses phylogeny classification, consisting of a single, hierarchically organised list that is compatible with short- and long-read data to identify microbial communities [109]. The NCBI taxonomy database also supplies the hierarchical taxon tree and standard nomenclature used by databases like GenBank, DNA Data Bank of Japan (DDBJ), and European Nucleotide Archive (ENA) that collectively form the International Nucleotide Sequence Database Collaboration (INSDC) which is adopted by most metagenomic classifiers. The SILVA database is a quality-controlled database of aligned rRNA sequences of bacteria, eukaryotes, and archaea. This database features regular releases that are made available rather than through continuous updates. As a result, the SILVA database has been highlighted as an ideal reference for high-throughput classification of NGS data for 16S rRNA approaches whereas the utilisation of INSDC or NCBI (home of GenBank) are recommended for metagenomic investigations [110, 111].

#### Workflows

Regardless of the methodological techniques, technology, and platform selected, all bioinformatic workflows begin with quality checks of raw data. The development of operational taxonomic units (OTUs) or amplicon sequence variants (ASVs) through read assignment appears in almost every 16S bioinformatic work for microbial classification. OTUs are formulated to enable sequencing errors to be distinguished from real nucleotide data. The formation of OTUs occurs via clustering reads on a pre-set identity threshold (commonly 97%), to repair sequencing errors [112]. Whilst OTUs were the most common throughout this review, the use of ASVs has overtaken the use of OTUs and also shown to significantly benefit direct compatibility between studies without the need for reanalysis of data with alpha diversity assessments influenced depending on the method used [113].

A metagenomic workflow can perform either read-based taxonomic classification or assembly based taxonomic classification which impact microbial identification differently [114]. The clustering process required in an assembly-based process or 16S approach can be bypassed by the trimmed reads being aligned to a known database of bacterial sequences for taxonomy. However, read-based taxonomic classification is commonly performed when handling well researched organisms with relatively small metagenomes and has shown to gather more functional annotation than an assembly-based approach. Assembly-based taxonomic classification, however, essentially group reads into specific genomes which are then assigned to bacteria and classified accordingly through reconstructing microbial genomes. This process has a superior performance than read-based taxonomic assignments and is more favourable for longer read lengths [114–117].

Studies that utilise short-read technology tend to use bioinformatic tools such as QIIME/QIIME2, USSEARCH, and DADA2 software for filtering, data quality parameters, and read clustering. QIIME/QIIME2 software was the most consistent tool used in data trimming, read clustering, chimera identification, and deletion and clustering of reads into OTUs for short read technology with the use of QIIME or QIIME2 dependent on the time of data analysis as QIIME2 superseded QIIME [13, 35, 40, 41, 49, 83]. Filtering, trimming, and quality checking are essential in the bioinformatic pipeline as raw reads are filtered for overall low quality, adapter sequences, low quality 5′ and 3′ tails, and contaminants, which are later removed from the data to provide more accurate and precise results. Chimeric sequences are incomplete remnants of previous PCR, with identification and removal of these essential in any bioinformatic pipeline [112]. Read clustering assembles the data into groups and, using specific statistics, reconstructs the genome and following this clustering, taxonomic assignment can occur. The taxonomic assignment entails a taxon of each sequence identified for a particular tool to match the sequence to a known database [112]. OTU assignment involves the use of QIIME, QIIME2, and Ribosomal Database Classifier (RDP). Some studies specifically include the Recombination Detection Program, which employs a pairwise scanning approach to analyse and identify recombinant DNA amongst a group of aligned sequences [118]. Finally, R studio and various R packages continue to provide the most popular tools used for data analysis across both short- and long-read sequencing outputs [12, 26, 36, 37, 39, 53, 67, 68, 71]. R studio provides a standard tool utilised in both technology studies for graphical taxonomic representation and has been demonstrated as advantageous in data analysis and taxonomic datasets.

Interestingly, there remains a lack of studies using mock communities with which to compare findings, ultimately making the results less robust for both short- and long-read technologies. The use of a mock microbial community for sequencing protocols and bioinformatic methodologies alongside unknown samples is necessary to validate the accuracy of methods selected. Synthetic mock community standards should be included in studies investigating microbiomes to identify potential biases and increase robustness [75].

Long-read sequencing data is typically analysed using Oxford Nanopore Technologies (ONT) software, such as the cloud-based data-analysis platform EPI2ME, and the base calling software GUPPY. Unlike short-read technologies, nanopore sequencing requires base-calling either during sequencing or following sequencing to allow for data output [119, 120]. QIIME software was developed for short-read length data analysis and its associated technologies and is not compatible with long-read data that generated from nanopore sequencing [121]. As such the use of bioinformatic tools for nanopore sequencing data analysis need to be specifically developed to accurately interpret long-read length data for accurate community identification.

#### Data availability and deposition

Reproducibility and repeatability of results is essential in microbiome investigations to validate a study’s findings and assess the consistency of results. To evaluate reproducibility and repeatability, however, datasets require re-analysis by researchers which can only be achieved if studies publish raw data or publish raw data into a microbiome data repository [122]. Whilst deposition of raw data into repositories like the International Nucleotide Sequence Database Collaboration (INSDC) centre are positive steps to increasing raw data availability across microbiome investigations, the data deposited is not integrated or processed within the INSDC centre due to a lack of funding [122, 123]. Due to the lack of data integration and processing after data deposition, the sequences have no reusability beyond the scope of the original investigation and unable to be repeated and reproduced within different studies. The lack of raw data available for re-analysis hinders meta-analysis and systematic reviews attempting to compare different methodological techniques and technologies within microbiome investigations due to the minimal consistency in methods. Additionally, tools that cause high variability within analysis protocols—like bioinformatic workflow and database selection—cannot be accurately compared. For example, the determination of QIIME vs. QIIME2 accuracy cannot be determined without comparing re-analysed data [123].

Whilst a comprehensive microbiome databank remains the ideal standard for data deposition in microbiome research, the lack of funding presents a significant barrier to its realisation. In the interim, ensuring that all raw microbiome data are made publicly available is essential for enhancing data reproducibility and repeatability, thereby facilitating more robust benchmarking and comparison across different methodological approaches.

## Conclusion

This study highlighted the methodological approaches across the literature used for DNA sequencing of the genital tract microbiome for women undergoing fertility treatment. Unfortunately, no uniformity was found in the reported DNA extraction techniques, primer design, and chosen hypervariable regions of the NGS approaches. However, the choice of bioinformatic pipeline used in these studies demonstrated that QIIME and QIIME2 software were the most commonly used tools in short-read sequencing analysis. It is important to note that no clear differences were observed in sequencing methodologies or DNA extraction approaches based on female reproductive tract anatomical sites. This highlights a potential limitation and an area for future investigation, as understanding the differences could lead to more tailored and effective microbial assessments.

This review recommends the application of nanopore sequencing in microbiome investigations, to increase sequencing accuracy, precision, and comparability between different studies using an assembly-based metagenomics approach with INSDC or NCBI (home of GenBank) databases. This study also identified a limited number of studies that used nanopore sequencing to assess vaginal, endometrial, uterine, or cervical microbiomes in women undergoing fertility treatment. In conclusion, future studies are required to develop a uniform and robust methodology for accurate and precise microbial representation for use in these samples. This could include standardised protocols for sampling, DNA extraction and bioinformatics, and development of a publicly available data repository for benchmarking as well as longitudinal studies to track changes in the microbiome over time.

## Declarations

We confirm that the manuscript has been read and approved by all named authors and that there are no other persons who satisfied the criteria for authorship but are not listed. We further confirm that the order of authors listed in the manuscript has been approved by all of us. We confirm that we have given due consideration to the protection of intellectual property associated with this work and that there are no impediments to publication, including the timing of publication, with respect to intellectual property. In so doing, we confirm that we have followed the regulations of our institutions concerning intellectual property. We understand that the corresponding author is the sole contact for the Editorial process (including Editorial Manager and direct communications with the office). He is responsible for communicating with the other authors about progress, submissions of revisions and final approval of proofs. We confirm that we have provided a current, correct email address which is accessible by the corresponding author.
